# Risk for mental illness and family composition after migration to Sweden

**DOI:** 10.1371/journal.pone.0251254

**Published:** 2021-05-07

**Authors:** Elisabeth Mangrio, Karin Sjöström, Mathias Grahn, Slobodan Zdravkovic

**Affiliations:** 1 Department of Care Science, Faculty of Health and Society, Malmö University, Malmö, Sweden; 2 MIM, Malmö Institute for Studies of Migration, Diversity and Welfare, Malmö University, Malmö, Sweden; 3 Unit for Safety and Security, Municipality of Malmö, Malmö, Sweden; The University of Hong Kong, HONG KONG

## Abstract

**Objectives:**

The aim of the present study is to determine how marital status and certain post-migration family structures are associated with the risk of mental illness among recently arrived Arabic- speaking refugees in Sweden.

**Methods:**

A cross-sectional study was conducted during 2015 and 2016. The study population was recruited by inviting all adult refugees who participated in the mandatory public integration support programme. All refugees that participated had received refugee status. A total of 681 of the invited participants returned the GHQ-12 questionnaires, through which the risk for mental illness was measured and only Arabic- speaking refugees (N = 638) were included in the analyses.

**Results:**

Marital status per se was not associated with a risk for mental illness. However, for the whole study sample there was a statistical significant odds ratio of 1.72 (95% CI 1.03–2.86). For male Arabic-speaking refugees with a spouse or child left behind in the home country there was a borderline significant increased risk for mental illness, odds ratio = 1. 87 (95% CI 0.99–3.56). The risk for female Arabic-speaking refugees was non-significant, odds ratio = 1.35 (95% CI 0.55–3.33).

**Conclusions:**

Arabic- speaking refugees who were separated from family members reported an increased risk for mental illness after arriving in the host country. Actions to facilitate family reunion after arriving as a refugee (in Sweden) seems to be an important factor to promote mental health among refugees.

## Introduction

Sweden has received a high number of refugees during the last 4–5 years [1]. In the southern part of Sweden, recent research indicate that around 40% of newly arrived refugee men and 30% newly arrived refugee women are suffering from mental illness [2]. A Swedish study on mental illness among asylum seekers and immigrants, found that around 43% were mentally ill [3]. The prevalence of depression, anxiety and risk of PTSD among refugees in Sweden, is around 50% [4]. In consistence, refugees in Europe are suffering from various different mental illnesses such as PTSD, anxiety and depression to a higher extent compared to the populations in the host countries [5]. The prevalence of mental illness differs dependent on which migrant group that is of focus, but in general, migrants with poor socioeconomic status such as unemployment or isolation contribute to higher rates of depression during resettlement [5]. In addition to war experiences, refugees experience stressful events both during and after migration. Three main causes of stress for refugees are recognized, the violent trauma that takes place in the home country which leads to the migration, the journey, and the adjustment to the life in a new and foreign country [6]. The mental health of refugees, which may be influenced by the migration experience, is defined as ‘pre-migration trauma and post-migration living difficulties’. The uncertainty of refugee status adds up to the above mentioned factors that may increase the risk for mental ill-health [7]. Prevalence of mental illness was higher among those who had not yet received residence permits [4].

Qualitative studies on refugees and their situation during resettlement in Sweden indicate that refugees mental health is influenced by the challenge of family separation after the arrival in Sweden. The worry about family memebers left behind may suggest an additional psychological burden together with settlement challenges such as poor living conditions, lack of social integration, cultural attitudes or unemployment [8] and adapting to a new culture and social life after arrival [9]. The change from a socio-centric society to an egocentric society as a risk factor for mental illness because of isolation, especially if there is separation from family members after arrival [3].

The recent increase in migration to Europe in general and to Sweden in particular [10], led to some temporary political changes of the right of refugees to be granted permanent residence permits based on family reunion [11]. This change in policy led to difficulties for refugees to be reunited with their families in Sweden, in the case the family had been separated during the migration process [11]. The policy change means in practice that the family need to proof an economic stability in order to be reunited with family members [11]. The willingness of countries to authorize the reunification of migrant families is supported by international human rights law [12], which clearly states that the family is a natural and fundamental group in society that is entitled to the protection of the state and society. If families are split, they are deprived of this fundamental right. Host countries usually recognize family reunification, as it has been shown to be an effective mechanism for helping migrants adapt to the new society [13]. Another study on family reunification and its impact for refugees and their mental health and integration [14], showed that family reunification was widely perceived to influence resettlement outcomes for the whole family. Through the reunification process, refugees consistently reported that only when the family became a unified entity, the health and wellbeing of its members could be properly supported. A large extent of the refugees in the study reported that they were feeling worried and unsettled because of their serious concerns about safety, welfare and health of family members left behind in their home countries [14]. Migrants general health and mental health in particular can thus be affected by whether or not a family is reunited after migration, which is of crucial importance for participating in the integration programme of the host country. Therefore, it is important to determine how marital status and post-migration family structures are associated with the risk for mental illness among recently arrived Arabic-speaking refugees in Sweden. The aim of the present study is to determine how marital status and certain post-migration family structures are associated with the risk of mental illness among recently arrived Arabic-speaking refugees in Sweden.

## Materials and methods

### Participants

Between February 2015 and February 2016, approximately 1,700 questionnaires were distributed to refugees who spoke Arabic or Dari and who participated in the mandatory public integration support programme to become established as residents in the Scania region of southern Sweden. In total, 681 questionnaires were returned by 93.7% of the Arabic-speaking refugees and, resulting in an overall response rate of approximately 39.5%. In the present study, only Arabic-speaking refugees were included (N = 638). All participants had arrived in Sweden on humanitarian grounds and were granted refugee status.

### Measures

In this study, the General Health Questionnaire (GHQ-12) [15] was used to examine the risk of mental illness. The scale is used to indicate risk for mental illness development and is used and well-suited for larger epidemiological studies. The instrument is a 12-item questionnaire with a four-point Likert scale measuring a person’s well-being, including mainly depressive symptoms, worry, sleep and cognitive functioning. Each item is a statement on a scale which goes from “not at all” to “much more than usual”. The questionnaire consists of both positive and negative questions. Positive questions (Items 1, 3, 4, 7, and 12) were scored inversely. Conventionally, the scale is made negative which means that a high value means low psychological well-being and a low value a high psychological well-being. We used a 0,0,1,1 scoring model. A score sum of ≥ 3 according to Goldberg’s original recommendation was used. The average time to complete the questions was two minutes.

Data collection was conducted through a self-administered questionnaire containing questions regarding health, sleep, level of education, well-being, living conditions, social relations, work, and access to healthcare among others and was translated by authorized translators and validated by civic and health communicators. A pilot study was conducted prior to the study with the aim of validating the questionnaire for comprehension of the included questions.

The family composition was based on two questions. The first question addressed marital status by the options ‘married’, ‘unmarried’, ‘divorced’, or ‘widow/widower’. The second question asked which family members the respondent was staying with in Sweden, giving the options of ‘partner’, ‘children below 18 years’, ‘all children below 18 years are here’, ‘do not have any children’, ‘with parents’, or being ‘single’. Based on this, the family composition was dichotomized into ‘incomplete’ and ‘complete’ family composition. The family composition was defined as complete if both spouses were present in Sweden along with all their children (if they have any). An incomplete family was defined as (a) at least one family member being outside Sweden.

The respondents’ educational level was categorized according to years of schooling: ‘low educational level (≤ 9 years)’, ‘medium educational level (10–12 years of school)’, and ‘high educational level (> 12 years)’. Age was measured as a continuous variable, but for descriptive statistics, it was divided into five categories: ages ‘18–34’, ‘35–44’, ‘45–54’, ‘55–64’, and ‘65–80’. Gender was divided into either ‘male’ or ‘female’.

Due to the aim of the present study, a number of respondents were excluded from the analyses. Individuals who were single with no children were excluded from the analysis (n = 197). A small number of individuals who were a single parent with or without children in Sweden were also excluded (n = 8 and n = 4, respectively). A total of 638 individuals were included in the analysis of marital status and 289 individuals were included in the analysis regarding family composition and the outcome of risk for mental illness.

### Analysis

Descriptive statistics were calculated as frequencies and percentages. Logistic regression was used to analyse the association between marital status or family composition and the risk for mental illness calculating odds ratios and 95% confidence intervals. Multivariate logistic regression was used for calculating adjusted odds ratios. Educational level, age, gender as well as marital status and family composition were considered as independent variables. The dichotomised GHQ-12 score (i.e. risk for mental illness) was used as the dependent variable. In the analysis of post-migration family structure, only individuals with a complete family composition versus an incomplete family–defined as having at least one family member outside of Sweden (partner or children)–were included in the analysis. Statistical analyses were performed with SPSS version 22.

### Ethical approval

The present study was approved by the Regional Ethical Committee in Lund, Sweden, approval number 2014/285. Before participation in the study, all informants were informed about the study. According to the ethical committee that approved this research, analysing of data is only permitted to be handled by a statistican involved in the research group. Therefore data cannot be shared.

## Results

The study included 638 respondents. See Table 1 for psychological and sociodemographic factors.

**Table 1:** Psychological and sociodemographic variables by gender in the total sample.

Variable		Males n (%)	Females n (%)	p-value†
Risk for mental illness (GHQ-12 ≥ 3)		199(48.8%)	78(46.2%)	0.57
No risk for mental illness (GHQ-12 ≤ 2)		209(51.2%)	91(53.8%)	
Educational level				0.60
	Low educational level (0–9 years)	102(23.8)	45(25.3)	
	Medium educational level (10–12 years)	98(22.9)	46(25.8)	
	High educational level (more than 12 years)	228(53.3)	87(48.9)	
Age				0.61
	18–34 years	226(51.8)	89(48.9)	
	35–44 years	113(25.9)	49(26.9)	
	45–54 years	66(15.1)	35(19.2)	
	55–64 years	29(6.7)	8(4.4)	
	65–80 years	2(0.5)	1(0.5)	
Family composition				<0.01
	Complete family (married/partner with all children in Sweden or no children	62(16.3)	39(26.4)	
	Incomplete family with one family member left outside of Sweden	155(40.7)	61(41.2)	
	Unmarried/widowed/divorced with all children in Sweden	1(0.3)	4(2.7)	
	Unmarried/widowed/divorced without all children in Sweden	4(1.0)	4(2.7)	
	Unmarried/widowed/divorced and no children	159(41.7)	40(27.0)	

†p-value< 0.05, two-tailed.

N = 638.

The adjusted analysis of marital status in Arabic-speaking refugees and risk for mental illness is presented in Table 2.

**Table 2:** The association between marital status and risk for mental illness.

Marital status	OR* (95 CI)	p-value
Married (Reference group)	1	
Unmarried	1.01 (0.69–1.48)	0.96
Divorced	1.28 (0.50–3.25)	0.60
Widow/Widower	1.15 (0.28–4.74)	0.92

*Adjusted for age, education and gender.

Analysing the association between marital status and risk for mental illness resulted in non-significant OR for being divorced 1.28 (95% CI 0.50–3.25) and for being widow/widower 1.15 (95% CI 0.28–4.74).

When adjusting for age, gender and educational level a higher but borderline significant male risk of mental illness was found, OR 1. 87 (95% CI 0.99–3.56) in an incomplete family composition. The risk of mental illness in women with an incomplete family structure was non-significant, OR 1.35 (95% CI 0.55–3.33). Independent of gender, there was a significantly higher odds ratio, OR 1.72 (95% CI 1.03–2.86), for incomplete families, as presented in Table 3.

**Table 3:** Logistic regression–family composition and the outcome of risk for mental illness, N = 289.

Family composition	Males	Females	All
	OR* 95%(CI)	OR* 95%(CI)	OR* 95%(CI)
Complete family–married/partner with all children in Sweden or not having children	1	1	1
Incomplete family with one family member left outside of Sweden (partner of children)	1.87 (0.99–3.56)	1.35 (0.55–3.33)	1.72 (1.03–2.86)

*Adjusted for age (continuous), gender and educational level.

Single refugee men and women were excluded from the logistic regression since not being the focus of this study. We found that 46,8% single refugee men had risk for mental illness and 52,5% refugee women had a risk for mental illness (data not shown).

## Discussion

Having an incomplete family with at least one family member outside of Sweden (partner or child) increased the risk of mental illness for the migrating Arabic-speaking refugees. Arabic- speaking refugees who arrived with an incomplete family, with either a missing wife/partner or a missing child/children, were more likely to experience mental illness than those with all family members in Sweden.

We know through recent European research from WHO, that refugees are suffering to a higher degree from different mental health issues such as PTSD, anxiety and depression [5]. A Swedish study by Lindgren et al found separation to be a common risk in migration and that it could be a mental health risk factor of refugees and asylum seekers [3]. A vast majority of Arabic-speaking asylum seekers reported worry about family members left behind as a substantial hardship to endure after arrival [16]. Another report from Sweden’s municipalities and county councils, indicate that Sweden`s more stringent refugee policies, characterized by closed borders, temporary residence permits and tougher requirements for family reunification, had some stabilizing effect on the country, its regions and municipalities [17]. On the other hand, the legislation and regulations, had some counter-productive effects on the mental illnesss among asylum seekers and refugees [17], which is in line with the results in the current study.

The findings of the present study suggest that there might be a difference in risk of mental illness depending on gender, considering the increased but borderline significant risk for males. Having this in mind, there may nevertheless be difference between males and females regarding responsibility for the protection and care of the family members and may as such be more prone to stress and risk for mental illness. Women who arrived with an incomplete family composition, were, to a greater extent, older and with worse mental health compared to the other groups (data not presented). Hence, mental ill- health could affect women and men differently [18, 19]. Women may have different ways of reacting to mental illness depending on country of origin [20]. This could partly explain why GHQ-12 did not capture all the psychological symptoms of the women in the present study. Furthermore, earlier research shows that women who are depressed tend to normalize experiences that physicians or other healthcare staff label as depressive symptoms. In addition to being depressed, this could also be associated with feelings of stigmatisation [21] and may influence the way that women in the current study responded to the questions in the GHQ-12.

In the present study, both single men and single women were excluded from the logistic regression analyses. However we found that 46,8% of the single refugee men and 52,2% of the single refugee women had a risk for mental illness (data not shown), probably caused by loneliness as the main cause for vulnerability.

Other stressors than migration may also explain an increased risk for mental illness in both single and married refugees. In a study investigating discrimination and mental illness among Arabs in Canada, an increase of discrimination was found after September 11, 2001, but no gender differences were found [22]. Further studies are needed regarding single refugee men and the risk for discrimination and mental illness [22].

In the present study, we found that marital status did not have any significant association with risk for mental illness. However, in a recently published paper from Sweden, results showed that those who were divorced or who experienced death of a spouse, were more at risk for all of the studied mental illnesses [23].

GHQ-12 was used for assessing mental illness, and this instrument is well validated for assessing risk factors for mental illness [15, 24]. It has been adopted by the World Health Organization (WHO) in a study of psychological disorders in primary healthcare and has been deemed the best validated among similar screening tools [15, 25, 26]. The version of the GHQ-12 used in this study has been shown to be a safe instrument of psychological health, and its validity characteristics are not considered to be influenced by gender, age or educational level [15]. However, to the best of our knowledge, GHQ-12 is not especially adapted for studies on migrants and refugees but has been previously used in this research field [27, 28]. According to Goldberg’s original recommendation, a score sum of ≥ 3 was used as a cut-off value for increased mental health risk. It could be discussed whether this cut-off score is appropriate for the population studied. However, the same scoring was used in another study that also focuses on refugees mental health [28].

The educational level of recently arrived migrants to Sweden is lower today than 15 years ago, contributing to increased challenges in introducing migrants to the labour market [29]. During the years 2010–2012, only 4% of the recently arrived migrants who participated in the Swedish settlement process had started to work [29]. Given that mental health is an important factor for integration and entry into the labour market in Sweden [29], family reunification may significantly contribute to the refugees’ integration.

The present study has some methodological issues that need to be addressed. The moderate external dropout rate may have a negative impact on the generalizability of the findings. Also, the fact that all participants were anonymous made any precise dropout analyses impossible and could be seen as a limitation. However, an approximate dropout analysis was performed by comparing the characteristics of the study participants with statistics from Sweden’s public emploment service. The analysis suggested that people with higher levels of education could be overrepresented in the present study [30]. With regard to the response rate, it is about the same or even higher as compared to other studies in the same field [31, 32]. The choice to only include refugees with complete and uncomplete family compositions was based on the fact that the group of single parents was very small and that single refugees without children were not considered as having a family of their own. This choice explained the high internal dropout rate observed. A further limitation is the way that family composition was asked for in the questionnaires which could be considered as narrow, since the importance of extended family members were not considered choice of covariates in the adjustment-model. The examined group in this study were, at the time of the GHQ-12 rating, in the beginning of the establishment process, which means that they did not work, which excluded employment as a significant covariate variable. Educational level as a covariate seemed to be a stronger variable since it has a scientifically known association with health outcome [33]. The inclusion of age and gender is based on previous research [34].

In summary, the main finding of this study is that the risk for mental ill-health seems to be related to an incomplete family composition among Arabic-speaking refugees as a whole.

## Conclusions

It may be seen as beneficial for the mental health of refugees to strive towards helping newly arrived refugee families to reunite with their family members. The findings appears to be of value for health professionals and social workers involved in the reception and care for migrants. The awareness of the higher risk for mental illness among this population especially among those who experienced separation and incomplete family composition seems to be of importance. Through this awareness, support could be given individually and with a focus of newly arrived refugees with a family separation trauma.
